# Effect of egg irradiation on development and sterility of wild-type and *Wolbachia* trans-infected *Aedes aegypti* mosquito vectors

**DOI:** 10.1371/journal.pone.0333297

**Published:** 2025-09-25

**Authors:** Pattamaporn Kittayapong, Suwannapa Ninphanomchai, Parinda Thayanukul, Wanitch Limohpasmanee

**Affiliations:** 1 Center of Excellence for Vectors and Vector-Borne Diseases, Faculty of Science, Mahidol University at Salaya, Nakhon Pathom, Thailand; 2 EcoHealth Research Center, Go Green Co., Ltd., Chachoengsao, Thailand; 3 Department of Biology, Faculty of Science, Mahidol University, Bangkok, Thailand; 4 Thailand Institute of Nuclear Technology, Ministry of Higher Education, Research and Innovation, Nakhon Nayok, Thailand; National Taiwan Ocean University, TAIWAN

## Abstract

Sterile Insect Technique (SIT), Incompatible Insect Technique (IIT) or a combination of the two has become alternative promising vector control approaches. In order to apply these approaches, the targeted mosquitoes need to be sterilized and released. So far, the irradiation of mosquitoes has been conducted at the pupae or adult stages. In this study, we investigated the possibility of applying X-ray irradiation at the egg stage and also assessed the effect on the development and sterility of both wild-type and *Wolbachia* trans-infected *Aedes aegypti* mosquito vectors. The eggs of both wild-type and *Wolbachia* trans-infected lines were irradiated using X-ray at the doses of 1, 3, 5 and 7 Gy. Development of immature stages was observed. For wild-type *Ae. aegypti*, X-ray irradiation at the doses from 3 Gy decreased the development of the first-instar larvae and increased the development of the third-instar larvae but there was no effect on pupae. For *Wolbachia* trans-infected ones, a irradiation dose as low as 1 Gy could increase the development of the forth-instar larvae while an irradiation dose of 7 Gy induced significantly high mortality to the pupae (*p* < 0.05). To assess sterility, males and females that emerged from irradiated eggs were mated with the non-irradiated ones. Our results showed that an irradiation dose of 7 Gy significantly caused more than 90% sterility in both wild-type males and females (*p* < 0.05). However, this irradiation dose could be reduced to 5 Gy to sterilize both males and females infected with *Wolbachia*. Our findings revealed, for the first time, that applying a low-dose X-ray irradiation at the egg stage could sterilize both wild-type and *Wolbachia* trans-infected *Ae. aegypti* when they become adults. Egg irradiation could make the implementation of SIT, IIT or combined SIT/IIT for vector control much more feasible as the sterile eggs are easier to distribute and operate when compared to other developmental stages of mosquitoes.

## Introduction

*Aedes aegypti* is a widespread and invasive mosquito species that is highly abundant in tropical countries [1]. Significant public health diseases, such as dengue, chikungunya and Zika, are transmitted by *Ae. aegypti* mosquitoes [2,3]. Currently, there are no highly efficient vaccines available for protection of dengue, chikungunya, and Zika diseases but several vaccine candidates are undergoing evaluation [4]. Regular immature surveillance and implementation of appropriate control measures have been recommended by WHO for vector control of *Aedes* mosquitoes [5]. However, challenges in insecticide resistance and treating small larval breeding sites have been highlighted [4]. Over the past decade, several technologies have been developed to strengthen the conventional vector control methods [6], i.e., the release of genetically modified mosquitoes (GMM) or transgenic males to reduce the target populations [6–9], the exploit of cytoplasmic incompatibility induction properties of *Wolbachia* by the incompatible insect technique (IIT) to enable the production of non-viable eggs [6,10,11], and the release of insects sterilized by radiation, known as the sterile insect technique (SIT), in order to reduce the reproduction of natural populations of the same species [6,12]. Because of social concerns, cultural acceptance, and regulatory approval, the use of some methods, such as GMM and IIT, for vector control has encountered a number of challenges, therefore, the radiation-based approach has been considered as the most feasible and safest solution [6].

Radio-sensitivity varies with age or age within the life stage [13] and thus different developmental stages among *Aedes* mosquitoes are not equally affected by radiation [14]. However, due to great variations in life cycle, life span, and exposure pathway, interaction between radiation and a wide range of mosquito species is not very well understood [15,16]. On the other hand, production of sterile males for release through application of the SIT approach has been mainly obtained by irradiating pupae [17–19]. To date, only one study has been conducted to compare the sensitivity of immature stages of *Ae. aegypti* with radiation, but that study focused on gamma radiation [20]. The use of gamma radiation has become problematic because of government regulation, supply, usage and disposal of radioactive isotopes [19,21]; as a result, an adequate alternative technology without the security risk, such as X-ray technology, has been urgently needed [21]. X-ray is among the ionizing radiation sources that have been utilized in sterile insect releasing program [22] and X-ray based sterilization has been studied for a variety of insect pests including mosquitoes [21,23–26]. However, only X-ray irradiation of pupae [23–25,27] or adults [26] has been studied. To date, no X-ray studies have been conducted on eggs or larvae of *Ae. aegypti* for comparison. Radiation has an effect on each developmental life cycle of *Ae. aegypti* [20], and the impact on each life history trait of the species varies with dosage [15].

In this study, we aimed to investigate the effect of X-ray irradiation at the egg stage on the development and sterility of *Wolbachia* trans-infected and uninfected *Aedes aegypti* mosquito vectors. Our findings should provide useful information on optimal dosage for radiation-induced sterility of *Ae. aegypti* at the egg stage which is most appropriate and feasible for SIT, IIT or combined SIT/IIT implementation for vector control.

## Materials and methods

### Ethics consideration

The experiments were reviewed and approved by the Faculty of Science, Mahidol University-Institutional Animal Care and Use Committee (MUSC-IACUC) (MUSC64-044-593).

### Mosquito colony and rearing

Both wild-type *Wolbachia*-uninfected and *w*AlbB *Wolbachia* trans-infected *Aedes aegypti* mosquito colonies were used in the present study. A wild-type *Wolbachia*-uninfected *Ae. aegypti* mosquito colony was originally established from mosquito eggs, which were collected by using ovitraps placed in several households in communities in Chatuchak District, Bangkok. A *w*AlbB *Wolbachia* trans-infected *Ae. aegypti* colony was obtained by direct microinjecting *w*AlbB *Wolbachia* into wild-type *Ae. aegypti* female mosquitoes from the Chatuchak colony described above. The *w*AlbB *Wolbachia* strain was extracted from the *Ae. albopictus* colony originating from Plaeng Yao District, Chachoengsao Province. An establishment of *w*AlbB *Wolbachia* trans-infected *Ae. aegypti* mosquitoes was done using the method described by Ruang-areerate and Kittayapong [28]. The characteristics of the *w*AlbB *Wolbachia* trans-infected *Ae. aegypti* mosquitoes were demonstrated in Kittayapong et al. [29].

In the experiments, mosquitoes were reared in an aluminum mosquito rearing cage sized 30 cm x 30 cm x 30 cm. in a screened climatic control insectary at the Center of Excellence for Vectors and Vector-Borne Diseases (CVVD), Faculty of Science, Mahidol University at Salaya, Nakhon Pathom, Thailand, with 75 ± 2% relative humidity, 27 ± 2°C, and a photoperiod of L12:D12, and were fed with 10% sucrose solution. Males and females were allowed to mate for 2–3 days; then the females were fed with pig blood by using the Hemotek membrane feeding system (Hemotek Ltd., UK) for 3–4 consecutive days after mating. The blood, obtained from a qualified slaughterhouse, was treated with 10% of EDTA (SCHARLAU, Spain) as an anticoagulant. Egg papers were placed in the containers half-filled with water inside the cage 1–3 days following blood-feeding. After 3–4 days, the egg papers were then collected, dried for 1–3 days at room temperature, and placed on a plastic tray prior to the irradiation process.

### Irradiation and sex separation procedure

In this experiment, a total of 2,000 *Wolbachia* uninfected *Ae. aegypti* eggs, stored not more than one month, were counted and separated into 5 portions of 400 eggs. Each portion of eggs was placed on a paper and then transferred into a plastic container volume 1,038.69 cm^3^ (diameter 11.5 cm) covered with a screened lid. One portion of eggs was used as a control. Four portions of eggs were transported by an air-condition car from the Center of Excellence for Vectors and Vector-Borne Diseases (CVVD), Faculty of Science, Mahidol University at Salaya, Nakhon Pathom Province to the Thailand Institute of Nuclear Technology (TINT) (Public Organization), Ministry of Higher Education, Science, Research and Innovation in Nakhon Nayok Province for irradiation. The distance from CVVD, where the rearing facility was located, to TINT was about 100 km or 3 hours by car for a round-trip. Irradiation was conducted by the experienced and trained staff of TINT.

Four portions of 400 eggs were irradiated with X-ray irradiator model RS 2400 (Rad Source Technologies, Inc., USA) at the irradiation doses of 1 Gy, 3 Gy, 5 Gy and 7 Gy respectively. For the RS 2400 setting, the irradiator basically auto-generated the irradiation time based on parameters that were formerly established by the manufacturer. In general, the irradiator was operated under precise settings: power of 145 Kev, current of 37.5 mA, and a dose rate of 0.24 Gy per second or 14.63 Gy per minute. Therefore, in this study, the irradiation time for 1 Gy, 3 Gy, 5 Gy, and 7 Gy was auto-generated as 4.17 seconds, 12.50 seconds, 20.83 seconds, and 29.17 seconds respectively.

### Mosquito development process

After irradiation, eggs were transported from TINT back to CVVD. Then they were hatched in deionized water and newly emerged larvae were transferred into a plastic tray sized 32 cm x 42 cm x 5 cm with a total of 100 larvae per tray. Larval diets were provided at an amount between 0.5–2.0 g per day according to the developmental stage. Dead larvae were removed by using a dropper and the number of dead larvae was counted and recorded daily. When larvae developed into pupae, male and female pupae were sex separated using the local pupal sex separator modified from the larval-pupal sex separator (John Hock Co., Ltd., USA). Then they were separately transferred into a mosquito cage sized 20 cm x 20 cm x 20 cm. The number of non-emerged male and female pupae was counted and recorded. After pupae became adults, 10% of sucrose solution was provided inside the mosquito cage.

### Sterility test

After emergence, *Wolbachia* uninfected *Ae. aegypti* aged 2–3 days were cross-mated according to the following mating pairs: 1) males emerged from irradiated eggs and non-irradiated females (IR Non-WolB M x Non-IR Non-WolB F); 2) females emerged from irradiated eggs and non-irradiated males (IR Non-WolB F x Non-IR Non-WolB M); and 3) non-irradiated males and non-irradiated females (Non-IR Non-WolB M x Non-IR Non-WolB F). Mosquitoes were allowed to mate for 2–3 days and then blood feeding was provided. Blood-fed females were individually transferred into a small plastic cup and the condition for oviposition was provided. Females were allowed to lay eggs for 3–5 days and the number of eggs laid per female was counted and recorded. Eggs were hatched as previously described and the number of hatched eggs was counted and recorded for assessment of sterility. The induce sterility (IS) was assessed in order to evaluate the effect of sterility, and it was calculated as 100% minus the residual fertility value, which was calculated as IS = 100%–(Ho/Hn) where Ho was the mean egg hatch rate of treatment cages, and Hn was the mean egg hatch rate of fertile control cages [24]. Four replicates were conducted for each experiment.

The same experiments were conducted on *w*AlbB *Wolbachia* trans-infected *Ae. aegypti* with three cross-mating pairs as follows: 1) males emerged from irradiated eggs and non-irradiated females (IR WolB M x Non-IR WolB F); 2) females emerged from irradiated eggs and non-irradiated males (IR WolB F x Non-IR WolB M); and 3) non-irradiated males and non-irradiated females (Non-IR WolB M x Non-IR WolB F).

### Statistical analysis

Data was entered and cleaned using Microsoft Office Excel 2016 and statistical analysis was performed using SPSS 18.0 (Mahidol University License, SPSS Inc., Chicago, USA). Numbers of eggs, larvae, pupae, and egg hatch rate were analyzed by using paired-sample t-test; and induced sterility (IS) was analyzed and compared with a theoretical value of 100 (IS = 100 when the male mosquito is completely sterile) [29] by using one sample t-test. P-values of less or equal to 0.05 were considered significant.

## Results

### Development of wild-type *Aedes aegypti* after being irradiated at the egg stage

**Fig 1:**
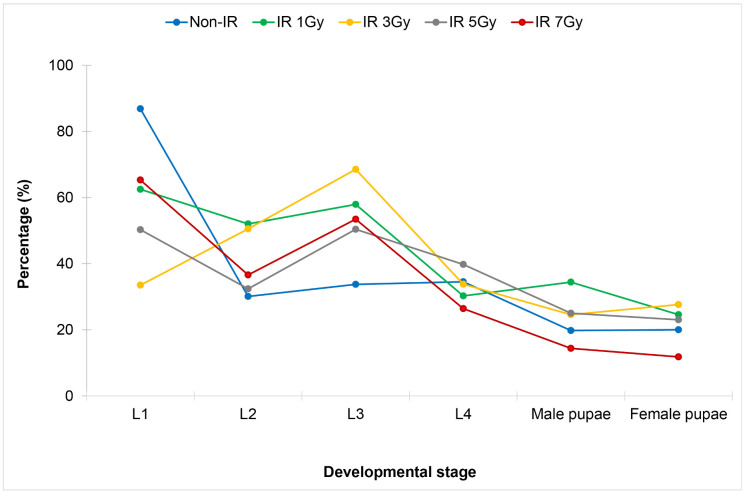
Comparison of larval and pupal development of wild-type *Wolbachia* uninfected *Aedes aegypti* after eggs being irradiated with X-ray at the irradiation doses of 1 Gy, 3 Gy, 5 Gy and 7 Gy.

Results showed that X-ray irradiation of the wild-type *Wolbachia* uninfected *Ae. aegypti* eggs with the irradiation doses from 3 Gy to 5 Gy significantly decreased the development of the first instar larvae, whereas the irradiation doses from 3 Gy to 7 Gy significantly increased the development of the third instar larvae when compared to those of the controls (Fig 1, Table 1). When larvae developed into pupae, late development or high mortality of pupae was observed at the irradiation dose of 7 Gy when compared to other irradiation doses or the controls; but this difference was not statistically significant (*p *< 0.05).

**Table 1:** Comparison of larval and pupal development of wild-type *Wolbachia* uninfected *Aedes aegypti* after eggs being irradiated with X-ray at the irradiation doses of 1 Gy, 3 Gy, 5 Gy and 7 Gy.

Variables	N	No. L1 larvae (Mean ± SD)	No. L2 larvae (Mean ± SD)	No. L3 larvae (Mean ± SD)	No. L4 larvae (Mean ± SD)	No. male pupae (Mean ± SD)	No. female pupae (Mean ± SD)
Non-IR Non-WolB	400	86.85 ± 27.01	30. 07 ± 29.24	33.73 ± 20.97	34.53 ± 23.32	19.75 ± 7.11	20.00 ± 15.63
IR Non-WolB-1 Gy	400	62.50 ± 16.58	52. 00 ± 20.89	57. 92 ± 21.06	30.25 ± 12.04	34.40 ± 20.23	24. 56 ± 15.13
IR Non-WolB-3 Gy	400	33.50 ± 26.42*	50.50 ± 15.86	68.50 ± 18.54*	33.75 ± 22.04	24.57 ± 5.94	27.63 ± 18.23
IR Non-WolB-5 Gy	400	50.25 ± 26.42*	32. 38 ± 25.53	50.38 ± 26.16*	39.77 ± 29.50	25.00 ± 20.48	23.00 ± 18.59
IR Non-WolB-7 Gy	400	65.29 ± 34.99	36.57 ± 28.57	53.43 ± 26.87*	26.38 ± 20.71	14.36 ± 16.24	11.80 ± 10.55

*Significant difference at *p* < 0.05.

### Sterility of wild-type *Aedes aegypti* after being irradiated at the egg stage

**Fig 2:**
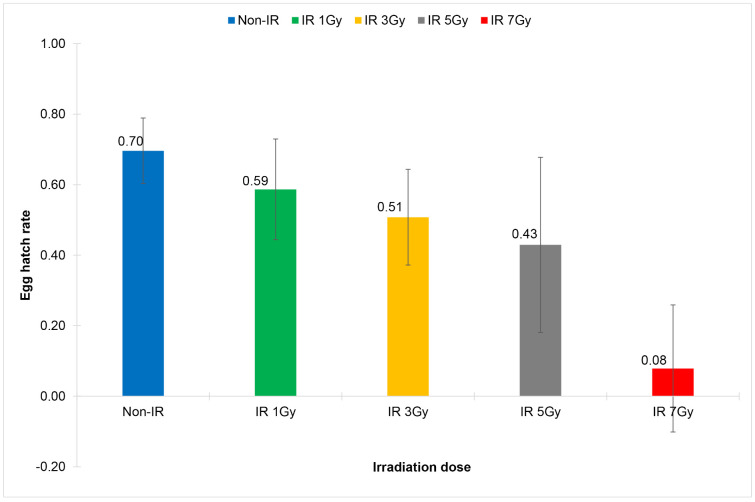
Comparison of egg hatch rates of the cross-mating pairs of wild-type *Wolbachia* uninfected *Aedes aegypti*: males emerged from irradiated eggs and non-irradiated females (IR Non-WolB ♂ x Non-IR Non-WolB ♀). Eggs were irradiated with X-ray at the irradiation doses of 1 Gy, 3 Gy, 5 Gy and 7 Gy.

In terms of the sterility of males emerging from irradiated eggs, it was found that the irradiation doses starting from 3 Gy onward significantly reduced the total number of eggs and the total number of hatched eggs of the cross-mating pairs: males emerged from irradiated eggs and non-irradiated females (IR Non-WolB M x Non-IR Non-WolB F), with a significant reduction of egg hatch rates at the irradiation doses starting from 1 Gy onward (0.59 ± 0.14 vs 0.70 ± 0.09, df = 29, t = 3.808, *p* = 0.001) (Fig 2, Table 2). However, males became nearly complete sterile at the irradiation dose of 7 Gy (IS = 99.92; 0.08 ± 0.18 vs 0.70 ± 0.09, df = 16.693, t = 29, *p* = 0.000) (Fig 2, Table 2).

**Table 2:** Comparison of total eggs, total hatched eggs, egg hatch rates, and induced sterility of the cross-mating pairs of wild-type *Wolbachia* uninfected *Aedes aegypti*: males emerged from irradiated eggs and non-irradiated females (IR Non-WolB ♂ x Non-IR Non-WolB ♀). Eggs were irradiated with X-ray at the irradiation doses of 1 Gy, 3 Gy, 5 Gy and 7 Gy.

Variables	N	Total eggs (Mean ± SD)	Total hatched eggs (Mean ± SD)	Egg hatch rates (Mean ± SD)	Induced Sterility (IS) (95%CI)
Non-IR Non-WolB ♂	120	3,384 (112.80 ± 11.87)	2,349 (78.30 ± 11.62)	0.70 (0.70 ± 0.09)	19.97 (−84.01 - −76.05)*
IR Non-WolB ♂ 1 Gy	120	3,047 (101.57 ± 33.86)	1,720 (57.33 ± 20.62)	0.59 (0.59 ± 0.14)*	32.55 (−73.57 - −61.32)*
IR Non-WolB ♂ 3 Gy	120	2,540 (84.67 ± 28.51)*	1,310 (43.67 ± 20.34)*	0.51 (0.51 ± 0.14)*	41.62 (−64.20 - −52.56)*
IR Non-WolB ♂ 5 Gy	120	2,192 (73.07 ± 47.79)*	1,155 (38.50 ± 27.61)*	0.43 (0.43 ± 0.25)*	50.64 (−60.01 - −38.70)*
IR Non-WolB ♂ 7 Gy	120	283 (9.43 ± 24.59)*	131 (4.37 ± 11.93)*	0.08 (0.08 ± 0.18)*	90.94 (−16.80 - −1.32)*

*Significant difference at *p* < 0.05.

**Fig 3:**
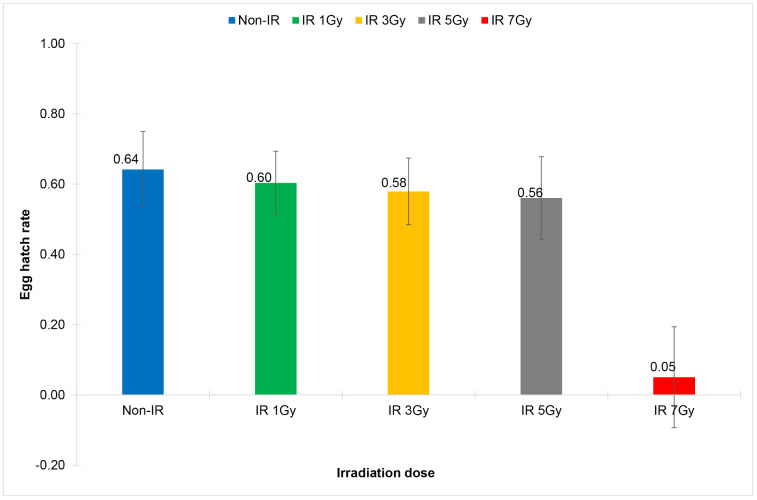
Comparison of egg hatch rates of the cross-mating pairs of wild-type *Wolbachia* uninfected *Aedes aegypti*: females emerged from irradiated eggs and non-irradiated males (IR Non-WolB ♀ x Non-IR Non-WolB ♂). Eggs were irradiated with X-ray at the irradiation doses of 1 Gy, 3 Gy, 5 Gy and 7 Gy.

For sterility of females emerged from irradiated eggs, it was found that the irradiation dose of 7 Gy significantly reduced the total number of eggs of the cross-mating pairs: females emerged from irradiated eggs and non-irradiated males (IR Non-WolB F x Non-IR Non-WolB M), and a significant reduction of egg hatch rates was observed at the irradiation doses starting from 3 Gy onward (0.58 ± 0.10 vs 0.64 ± 1.01, df = 29, t = 2.376, *p* = 0.024) (Fig 3, Table 3); and females became nearly complete sterile at the irradiation dose of 7 Gy (IS = 99.95; 0.05 ± 0.14 vs 0.64 ± 1.01, df = 29, t = 17.124, *p* = 0.000).

**Table 3:** Comparison of total eggs, total hatched eggs, egg hatch rates and induced sterility of cross-mating pairs of wild-type *Wolbachia* uninfected *Aedes aegypti*: females emerged from irradiated eggs and non-irradiated males (IR Non-WolB ♀ x Non-IR Non-WolB ♂). Eggs were irradiated with X-ray at the irradiation doses of 1 Gy, 3 Gy, 5 Gy and 7 Gy.

Variables	N	Total eggs (Mean ± SD)	Total hatched eggs (Mean ± SD)	Egg hatch rates (Mean ± SD)	Induced Sterility (IS) (95%CI)
Non-IR Non-WolB ♀	30	2,925 (97.50 ± 16.63)	1,859 (61.97 ± 12.07)	0.64 (0.64 ± 1.01)	24.47 (−80.25 - −70.81)*
IR Non-WolB ♀-1 Gy	30	2,591 (83.37 ± 16.33)	1,551 (51.70 ± 10.94)*	0.60 (0.60 ± 0.09)	28.99 (−74.96 - −67.06)*
IR Non-WolB ♀-3 Gy	30	2,647 (88.23 ± 23.54)	1,523 (50.77 ± 15.19)*	0.58 (0.58 ± 0.10)*	33.43 (−70.65 - −62.48)*
IR Non-WolB ♀-5 Gy	30	2,620 (87.33 ± 27.06)	1,483 (49.43 ± 18.34)*	0.56 (0.56 ± 0.12)*	34.02 (−71.13 - −60.82)*
IR Non-WolB ♀-7 Gy	30	384 (12.80 ± 34.51)*	136 (4.53 ± 13.20)*	0.05 (0.05 ± 0.14)*	94.22 (−11.95 - −0.40)

*Significant difference at *p* < 0.05.

To summarize, when wild-type *Wolbachia* uninfected eggs of *Ae. aegypti* were irradiated with X-ray, the irradiation dose of 7 Gy significantly induced more than 90% sterility of both males and females. Therefore, an irradiation dose of 7 Gy could be the optimum dose for irradiation of wild-type *Wolbachia* uninfected *Ae. aegypti* eggs.

### Development of *Wolbachia* trans-infected *Aedes aegypti* after being irradiated at the egg stage

**Fig 4:**
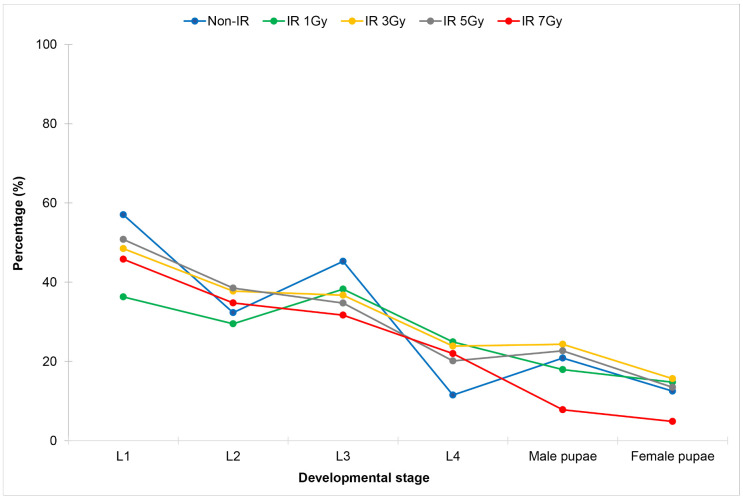
Comparison of larval and pupal development of *Wolbachia* trans-infected *Aedes aegypti* after eggs being irradiated with X-ray at the irradiation doses of 1 Gy, 3 Gy, 5 Gy and 7 Gy.

When *Wolbachia* trans-infected *Ae. aegypti* eggs were irradiated, it was found that the irradiation dose from 1 Gy onward significantly increased the development of the fourth-instar larvae when compared to those of the controls (Fig 4, Table 4). When larvae developed into pupae, significantly high mortality of both male and female pupae was observed at the irradiation dose of 7 Gy when compared to those of the controls.

**Table 4:** Comparison of larval and pupal development of *Wolbachia* trans-infected *Aedes aegypti* after eggs being irradiated with X-ray at the irradiation doses of 1 Gy, 3 Gy, 5 Gy and 7 Gy.

Variables	N	No. L1 larvae (Mean ± SD)	No. L2 larvae (Mean ± SD)	No. L3 larvae (Mean ± SD)	No. L4 larvae (Mean ± SD)	No. male pupae (Mean ± SD)	No. female pupae (Mean ± SD)
Non-IR WolB	400	57.00 ± 40.60	32.31 ± 29.55	45. 25 ± 35.35	11.50 ± 7.49	20.83 ± 19.30	12.50 ± 6.92
IR WolB-1 Gy	400	36.28 ± 41.93	29.50 ± 29.22	38.25 ± 20.50	24.93 ± 14.48*	17.92 ± 14.72	14.75 ± 12.25
IR WolB-3 Gy	400	48.45 ± 42.91	37.73 ± 34.44	36.70 ± 27.55	23.86 ± 11.12*	24.33 ± 12.91	15.64 ± 9.69
IR WolB-5 Gy	400	50.80 ± 43.51	38.50 ± 34.52	34.73 ± 26.10	20.11 ± 12.00*	22.67 ± 9.63	13.46 ± 10.16
IR WolB-7 Gy	400	45.80 ± 43.74	34.76 ± 32.06	31.67 ± 24.96	21.96 ± 11.42*	7.80 ± 7.97*	4.84 ± 3.66*

*Significant difference at *p* < 0.05.

### Sterility of *Wolbachia* trans-infected *Aedes aegypti* after being irradiated at the egg stage

**Fig 5:**
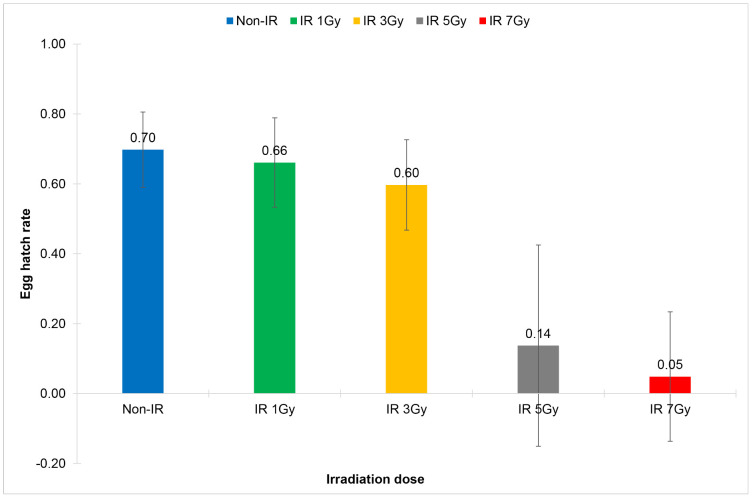
Comparison of egg hatch rates of the cross-mating pairs of *Wolbachia* trans-infected *Aedes aegypti*: males emerged from irradiated eggs and non-irradiated females (IR WolB ♂ x Non-IR WolB ♀). Eggs were irradiated with X-ray at the irradiation doses of 1 Gy, 3 Gy, 5 Gy and 7 Gy.

In terms of the sterility of males emerged from irradiated eggs, it was found that the irradiation dose of 1 Gy significantly reduced the total number of eggs and the total number of hatched eggs of the cross-mating pairs: males emerged from irradiated eggs and non-irradiated females (IR WolB M x Non-IR WolB F); and a significant reduction of the egg hatch rates was observed when the irradiation dose was at 3 Gy (0.60 ± 0.13 vs 0.70 ± 0.11, df = 29, t = 3.113, *p* = 0.004) (Fig 5, Table 5). Dramatically significant reduction of the egg hatch rates was observed when the irradiation doses were at 5 Gy (0.14 ± 0.29 vs 0.70 ± 0.11, df = 29, t = 10.691, *p* = 0.000) and at 7 Gy (0.05 ± 0.19 vs 0.70 ± 0.11, df = 29, t = 16.824, *p* = 0.000) (Fig 5, Table 5).

**Table 5:** Comparison of total eggs, total hatched eggs, egg hatch rates and induced sterility (IS) of the cross-mating pairs of *Wolbachia* trans-infected *Aedes aegypti*: males emerged from irradiated eggs and non-irradiated females (IR WolB ♂ x Non-IR WolB ♀). Eggs were irradiated with X-ray at the irradiation doses of 1 Gy, 3 Gy, 5 Gy and 7 Gy.

Variables	N	Total eggs (Mean ± SD)	Total hatched eggs (Mean ± SD)	Egg hatch rates (Mean ± SD)	Induced Sterility (IS) (95%CI)
Non-IR WolB ♂	120	3,009 (100.30 ± 30.51)	2,069 (68.97 ± 19.22)	0.70 (0.70 ± 0.11)	26.25 (−73.75 - −69.93)*
IR WolB ♂-1 Gy	120	2,277 (75.90 ± 21.57)*	1,480 (49.33 ± 14.60)*	0.66 (0.66 ± 0.13)	29.68 (−75.40 - −65.25)*
IR WolB ♂-3 Gy	120	2,147 (71.57 ± 22.65)*	1,256 (41.87 ± 14.44)*	0.60 (0.60 ± 0.13)*	36.48 (−68.64 - −58.39)*
IR WolB ♂-5 Gy	120	446 (14.87 ± 31.22)*	300 (10.00 ± 21.35)*	0.14 (0.14 ± 0.29)*	85.39 (−26.05 - −3.16)*
IR WolB ♂-7 Gy	120	169 (5.63 ± 17.22)*	72 (2.40 ± 9.14)*	0.05 (0.05 ± 0.19)*	94.83 (−12.53 - −2.19)

*Significant difference at *p* < 0.05.

**Fig 6:**
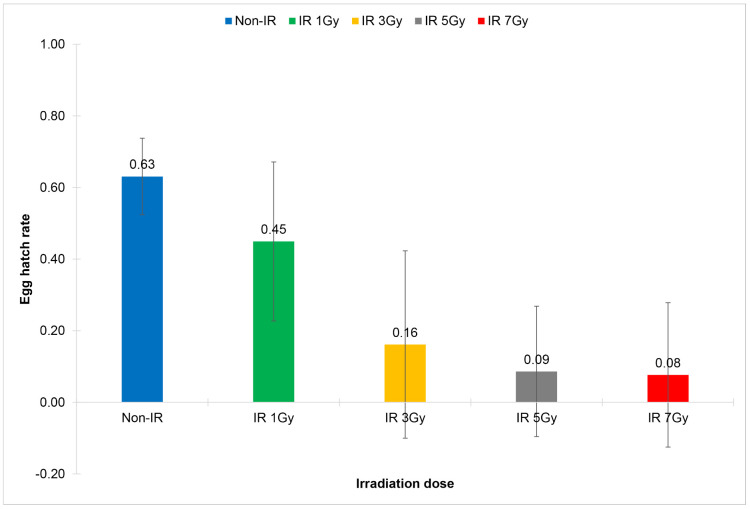
Comparison of egg hatch rates of the cross-mating pairs of *Wolbachia* trans-infected *Aedes aegypti*: females emerged from irradiated eggs and non-irradiated males (IR WolB ♀ x Non-IR WolB ♂). Eggs were irradiated with X-ray at the irradiation doses of 1 Gy, 3 Gy, 5 Gy and 7 Gy.

For sterility of females emerged from irradiated eggs, it was found that the irradiation dose of 1 Gy significantly reduced the total number of eggs and the total number of hatched eggs of the cross-mating pairs: females emerged from irradiated eggs and non-irradiated males (IR WolB F x Non-IR WolB M). A significant reduction of the egg hatch rates was observed when the irradiation doses were at 1 Gy (0.45 ± 0.22 vs 0.63 ± 0.11, df = 29, t = 3.940, *p* = 0.000) and at 3 Gy (0.16 ± 0.26 vs 0.63 ± 0.11, df = 29, t = 10.020, *p* = 0.000) (Fig 6, Table 6). Moreover, dramatically significant reduction of the egg hatch rates was observed or females became nearly complete sterile when the irradiation dose was at 5 Gy (IS = 99.91, 0.09 ± 0.18 vs 0.63 ± 0.11, df = 29, t = 14.707, *p* = 0.000) (Fig 6, Table 6).

**Table 6:** Comparison of total eggs, total hatched eggs, egg hatch rates and induced sterility (IS) of the cross-mating pairs of *Wolbachia* trans-infected *Aedes aegypti*: females emerged from irradiated eggs and non-irradiated males (IR WolB ♀ x Non-IR WolB ♂). Eggs were irradiated with X-ray at the irradiation doses of 1 Gy, 3 Gy, 5 Gy and 7 Gy.

Variables	N	Total eggs (Mean ± SD)	Total hatched eggs (Mean ± SD)	Egg hatch rates (Mean ± SD)	Induced Sterility (IS) (95%CI)
Non-IR WolB ♀	120	3,130 (104.33 ± 27.58)	1,944 (64.80 ± 16.43)	0.63 (0.63 ± 0.11)	26.67 (−77.80 - −68.86)*
IR WolB ♀-1 Gy	120	2,371 (79.03 ± 25.53)*	1,080 (36.00 ± 22.42)*	0.45 (0.45 ± 0.22)*	47.74 (−61.57 - −42.95)*
IR WolB ♀-3 Gy	120	1,997 (66.57 ± 38.91)*	419 (13.97 ± 23.53)*	0.16 (0.16 ± 0.26)*	81.21 (−29.76 - −7.81)*
IR WolB ♀-5 Gy	120	1,540 (51.33 ± 46.79)*	173 (5.77 ± 12.04)*	0.09 (0.09 ± 0.18)*	89.98 (−17.65. - −2.38)*
IR WolB ♀-7 Gy	120	361 (12.03 ± 25.35)*	141 (4.70 ± 12.88)*	0.08 (0.08 ± 0.20)*	91.09 (−17.37 - −0.46)*

*Significant difference at *p* < 0.05.

In summary, the X-ray irradiation dose of 7 Gy significantly induced male sterility, whereas the irradiation dose of only 5 Gy significantly induced female sterility. Therefore, an X-ray irradiation dose of 5 Gy could be the optimum dose for irradiation of *Wolbachia* trans-infected *Ae. aegypti* eggs as it could induce high level of sterility in males and nearly complete sterility in females.

## Discussion

In this study, we found that as X-ray irradiation dose increased, nearly complete sterility was progressively achieved in both wild-type and *Wolbachia* trans-infected *Ae. aegypti*. Lower dose of X-ray irradiation was sufficient to induce complete sterility in both wild-type and *Wolbachia* trans-infected *Ae. aegypti* when irradiation was done at the egg stage. An X-ray irradiation dose of 5 Gy and 7 Gy could be the optimum doses to induce nearly complete sterility in *Wolbachia* trans-infected and wild-type *Ae. aegypti* respectively. Irradiation-induced dominant lethal mutations in the germ cells were the main cause of male sterilization in the SIT approach [30]. However, a higher dose of radiation exposure could decrease male quality if the males were overdosed [31]. Our study showed that an increased irradiation dose could reduce male quality in both wild-type and *Wolbachia* trans-infected *Ae. aegypti*, since we observed that less eggs were laid by females that were previously mated with wild-type and *Wolbachia* trans-infected *Ae. aegypti* males when those two were exposed to higher doses of radiation. Therefore, optimizing the irradiation step to achieve maximum sterility while minimizing the somatic damage could significantly improve the quality of sterile *Ae. aegypti* mosquitoes [32].

Irradiation of different mosquito species at various stages of the life cycle showed increased or declined effects on adult life span including its subsequent generations [18,33]. Age had been shown to have an effect on radio-sensitivity, and the older the life stage, or the aging within the life stages, the more radio-resistant the insects became [13]. Treatment of early life stages of mosquitoes, together with environmental alterations, greatly influenced the lifespan of adult mosquitoes [15], so in order to reduce somatic damage, irradiation of insects at or near to the completion of their development, i.e., the late pupal and adult stages of mosquitoes, had been emphasized [14,15,19,23]. However, the optimum developmental stage for irradiation depended on many factors including ease of handling on a mass-production scale, logistics of the irradiation process (e.g., the need to irradiate large numbers of insects), competitiveness of the insects, release methodology, and costs [19]. In this study, we investigated the effect of irradiation at the egg stage on sterility of *Wolbachia* trans-infected and uninfected *Ae. aegypti*, in order to find an appropriate and more convenient method for mass production of sterile males prior to the release of sterile males for vector control.

So far, not much information has been available on the irradiation of eggs, especially for mosquitoes. Due to inefficient results in controlling *Aedes*-transmitted arboviral diseases, research on irradiation of the mosquito stages, beyond adults and immatures, was needed [34]. In this study, we observed a late pupation and high mortality in pupae when *Ae. aegypti* eggs were irradiated with X-ray at an irradiation dose of 7 Gy. Our results were supported by Tantawy et al. [35] who showed high mortality when eggs of the *Anopheles pharoensis* were irradiated with gamma radiation. Moreover, our studies showed that an irradiation dose of 7 Gy significantly induced more than 90% sterility in the wild-type *Wolbachia* uninfected *Ae. aegypti* males and females after being irradiated at the egg stage. In terms of *Wolbachia* trans-infected *Ae. aegypti*, the same irradiation dose of 7 Gy also induced sterility in males, and a lower dose of 5 Gy was sufficient to induce complete sterility in females. Our results were supported by the study of Akter and Khan [20] who found that low dose radiation from 1–10 Gy had significant effects on pupation and adult emergence when they were irradiated at the egg stage. In addition, our results coincided with the study of Furaki et al. [36] who observed a reduction in egg hatching, as well as in adult emergence, among flour beetles and almond moths when their eggs were exposed to UV irradiation. The same study concluded that damage to the surface tissues of the eggs by radiation could be fatal, especially at advanced stages of development [36].

Although pupae is the preferred stages to sterilize during SIT operations, due to limited pupation time, the distances for their distribution are limited to closer localities where the irradiation source is located [34]. Eggs can be distributed to logistically remote field sites where they can hatch, develop into adults, and fly out to compete with wild mosquitoes [37]. The cost-benefit of using the egg stage in field distribution is very promising in low-income endemic countries. Hence, the expansion of sterilized insect programs could be facilitated by using either irradiated eggs or *Wolbachia* vertical infection [34]. Our results showed the effect of low-dose X-ray irradiation on sterility of both male and female wild-type and *Wolbachia* trans-infected *Ae. aegypti* when eggs were irradiated, and how this approach could be applied for SIT operations for vector control in the field.

## Supporting information

S1 File Data used to generate Fig 1. (XLSX)

S2 File Data used to generate Fig 2. (XLSX)

S3 File Data used to generate Fig 3. (XLSX)

S4 File Data used to generate Fig 4. (XLSX)

S5 File Data used to generate Fig 5. (XLSX)

S6 File Data used to generate Fig 6. (XLSX)

S7 File Data used to generate Table 2. (XLSX)

S8 File Data used to generate Table 3. (XLSX)

S9 File Data used to generate Table 5. (XLSX)

S10 File Data used to generate Table 6. (XLSX)

## References

[1] Godoy RSM, Felix LDS, Orfanó A da S, Chaves BA, Nogueira PM, Costa BDA, et al. Dengue and Zika virus infection patterns vary among Aedes aegypti field populations from Belo Horizonte, a Brazilian endemic city. PLoS Negl Trop Dis. 2021;15(11):e0009839. doi: 10.1371/journal.pntd.0009839 34727099 PMC8562804

[2] Carrasquilla MC, Ortiz MI, León C, Rondón S, Kulkarni MA, Talbot B, et al. Entomological characterization of Aedes mosquitoes and arbovirus detection in Ibagué, a Colombian city with co-circulation of Zika, dengue and chikungunya viruses. Parasit Vectors. 2021;14(1):446. doi: 10.1186/s13071-021-04908-x 34488857 PMC8419972

[3] Chowdhury A, Modahl CM, Missé D, Kini RM, Pompon J. High resolution proteomics of Aedes aegypti salivary glands infected with either dengue, Zika or chikungunya viruses identify new virus specific and broad antiviral factors. Sci Rep. 2021;11(1):23696. doi: 10.1038/s41598-021-03211-0 34880409 PMC8654903

[4] Evans-Gilbert T. Vertically transmitted chikungunya, Zika and dengue virus infections: The pathogenesis from mother to fetus and the implications of co-infections and vaccine development. Int J Pediatr Adolesc Med. 2020;7(3):107–11. doi: 10.1016/j.ijpam.2019.05.004 33094137 PMC7567994

[5] Daniel Reegan A, Rajiv Gandhi M, Cruz Asharaja A, Devi C, Shanthakumar SP. COVID-19 lockdown: Impact assessment on Aedes larval indices, breeding habitats, effects on vector control programme and prevention of dengue outbreaks. Heliyon. 2020;6(10):e05181. doi: 10.1016/j.heliyon.2020.e05181 33043162 PMC7534600

[6] Gouagna LC, Damiens D, Oliva CF, Boyer S, Le Goff G, Brengues C, et al. Strategic approach, advances, and challenges in the development and application of the SIT for area-wide control of Aedes albopictus mosquitoes in Reunion Island. Insects. 2020;11(11):770. doi: 10.3390/insects11110770 33171885 PMC7695178

[7] Thomas DD, Donnelly CA, Wood RJ, Alphey LS. Insect population control using a dominant, repressible, lethal genetic system. Science. 2000;287(5462):2474–6. doi: 10.1126/science.287.5462.2474 10741964

[8] Carvalho DO, McKemey AR, Garziera L, Lacroix R, Donnelly CA, Alphey L, et al. Suppression of a field population of Aedes aegypti in Brazil by sustained release of transgenic male mosquitoes. PLoS Negl Trop Dis. 2015;9(7):e0003864. doi: 10.1371/journal.pntd.0003864 26135160 PMC4489809

[9] Alphey N, Coleman PG, Donnelly CA, Alphey L. Managing insecticide resistance by mass release of engineered insects. J Econ Entomol. 2007;100(5):1642–9. doi: 10.1603/0022-0493(2007)100[1642:mirbmr]2.0.co;2 17972643

[10] O’Connor L, Plichart C, Sang AC, Brelsfoard CL, Bossin HC, Dobson SL. Open release of male mosquitoes infected with a Wolbachia biopesticide: Field performance and infection containment. PLoS Negl Trop Dis. 2012;6(11):e1797. doi: 10.1371/journal.pntd.0001797 23166845 PMC3499408

[11] Sinkins SP. Wolbachia and cytoplasmic incompatibility in mosquitoes. Insect Biochem Mol Biol. 2004;34(7):723–9. doi: 10.1016/j.ibmb.2004.03.025 15242714

[12] Knipling EF. Possibilities of insect control or eradication through the use of sexually sterile males1. J Economic Entomol. 1955;48(4):459–62. doi: 10.1093/jee/48.4.459

[13] Yamada H, Maiga H, Juarez J, De Oliveira Carvalho D, Mamai W, Ali A, et al. Identification of critical factors that significantly affect the dose-response in mosquitoes irradiated as pupae. Parasit Vectors. 2019;12(1):435. doi: 10.1186/s13071-019-3698-y 31500662 PMC6734225

[14] Poda SB, Guissou E, Maïga H, Bimbile-Somda SN, Gilles J, Rayaisse J-B, et al. Impact of irradiation on the reproductive traits of field and laboratory An. arabiensis mosquitoes. Parasit Vectors. 2018;11(1):641. doi: 10.1186/s13071-018-3228-3 30558681 PMC6296153

[15] Shetty V, Shetty NJ, Harini BP, Ananthanarayana SR, Jha SK, Chaubey RC. Effect of gamma radiation on life history traits of Aedes aegypti (L.). Parasite Epidemiol Control. 2016;1(2):26–35. doi: 10.1016/j.parepi.2016.02.007 29988174 PMC5991819

[16] Singhal RK, Ajay K, Usha N, Reddy AVR. Evaluation of doses from ionising radiation to non-human species at Trombay, Mumbai, India. Radiat Prot Dosimetry. 2009;133(4):214–22. doi: 10.1093/rpd/ncp048 19339303

[17] Lees RS, Carvalho DO, Bouyer J. Potential impact of integrating the sterile insect technique into the fight against disease-transmitting mosquitoes. In Sterile Insect Technique. 2nd ed. CRC Press. 2021.

[18] Helinski MEH, Parker AG, Knols BGJ. Radiation-induced sterility for pupal and adult stages of the malaria mosquito Anopheles arabiensis. Malar J. 2006;5:41. doi: 10.1186/1475-2875-5-41 16700906 PMC1475870

[19] Helinski MEH, Parker AG, Knols BGJ. Radiation biology of mosquitoes. Malar J. 2009;8 Suppl 2(Suppl 2):S6. doi: 10.1186/1475-2875-8-S2-S6 19917076 PMC2777328

[20] Akter H, . SAK. Sensitivity of immature stages of dengue causing mosquito, Aedes aegypti (L.) to gamma radiation. J Entomol. 2014;11(2):56–67. doi: 10.3923/je.2014.56.67

[21] Haff R, Ovchinnikova I, Liang P, Mahoney N, Gee W, Gomez J, et al. X-Ray-based irradiation of larvae and pupae of the navel orangeworm (Lepidoptera: Pyralidae). J Econ Entomol. 2020;113(4):1685–93. doi: 10.1093/jee/toaa111 32556336

[22] Ayvaz A, Yilmaz S. Ionizing radiation disinfestation treatments against pest insects. Evol Ionizing Rad Res. InTech. 2015. doi: 10.5772/60923

[23] Bond JG, Osorio AR, Avila N, Gómez-Simuta Y, Marina CF, Fernández-Salas I, et al. Optimization of irradiation dose to Aedes aegypti and Ae. albopictus in a sterile insect technique program. PLoS One. 2019;14(2):e0212520. doi: 10.1371/journal.pone.0212520 30779779 PMC6380561

[24] Zhang D, Lees RS, Xi Z, Bourtzis K, Gilles JRL. Combining the sterile insect technique with the incompatible insect technique: III-Robust mating competitiveness of irradiated triple Wolbachia-Infected Aedes albopictus males under semi-field conditions. PLoS One. 2016;11(3):e0151864. doi: 10.1371/journal.pone.0151864 26990981 PMC4798476

[25] Yamada H, Parker AG, Oliva CF, Balestrino F, Gilles JRL. X-ray-induced sterility in Aedes albopictus (Diptera: Culicidae) and male longevity following irradiation. J Med Entomol. 2014;51(4):811–6. doi: 10.1603/me13223 25118413

[26] Rodriguez SD, Brar RK, Drake LL, Drumm HE, Price DP, Hammond JI, et al. The effect of the radio-protective agents ethanol, trimethylglycine, and beer on survival of X-ray-sterilized male Aedes aegypti. Parasit Vectors. 2013;6:211. doi: 10.1186/1756-3305-6-211 23866939 PMC3723957

[27] Hallinan E, Rai KS. Radiation sterilization of Aedes aegypti in nitrogen and implications for sterile male technique. Nature. 1973;244(5415):368–9. doi: 10.1038/244368a0 4583511

[28] Ruang-Areerate T, Kittayapong P. Wolbachia transinfection in Aedes aegypti: A potential gene driver of dengue vectors. Proc Natl Acad Sci USA. 2006;103(33):12534–9. doi: 10.1073/pnas.0508879103 16895998 PMC1567913

[29] Kittayapong P, Ninphanomchai S, Thayanukul P, Yongyai J, Limohpasmanee W. Comparison on the quality of sterile Aedes aegypti mosquitoes produced by either radiation-based sterile insect technique or Wolbachia-induced incompatible insect technique. PLoS One. 2025;20(2):e0314683. doi: 10.1371/journal.pone.0314683 39937795 PMC11819552

[30] Dyck VA, Hendrichs J, Robinson AS. Sterile Insect Technique: Principles and Practice in Area Wide Integrated Pest Management. Springer Netherlands. 2005.

[31] Diallo S, Seck MT, Rayaissé JB, Fall AG, Bassene MD, Sall B, et al. Chilling, irradiation and transport of male Glossina palpalis gambiensis pupae: Effect on the emergence, flight ability and survival. PLoS One. 2019;14(5):e0216802. doi: 10.1371/journal.pone.0216802 31086401 PMC6516675

[32] Yamada H, Maïga H, Kraupa C, Somda NSB, Mamai W, Wallner T, et al. Radiation dose-fractionation in adult Aedes aegypti mosquitoes. Parasite. 2023;30:5. doi: 10.1051/parasite/2023005 36762942 PMC9912927

[33] Abdel-Malek AA, Tantawy AO, Wakid AM. Studies on the eradication of Anopheles pharoensis Theobald by the sterile-male technique using cobalt-60. I. Biological effects of gamma radiation on the different developmental stages. J Econ Entomol. 1966;59(3):672–8. doi: 10.1093/jee/59.3.672 5932263

[34] Martínez-García EN, Díaz-González EE, Marina CF, Bond JG, Rodríguez-Rojas JJ, Ponce-García G, et al. Temporal Viability of Aedes aegypti and Aedes albopictus Eggs Using Two Hygroscopic Substances as Preservatives under a Sterile Insect Technique (SIT) Program in Southern Mexico. Insects. 2021;13(1):15. doi: 10.3390/insects13010015 35055859 PMC8780675

[35] Tantawy AO, Abdel-Malek AA, Wakid AW. Studies on eradication of Anopheles pharoensis by the sterile-male technique using cobalt-60. II. Induced dominant lethals in the immature stages. J Econ Entomol. 1966;59(6):1392–4. doi: 10.1093/jee/59.6.1392 5976112

[36] Faruki SI, Das DR, Khan AR, Khatun M. Effects of ultraviolet (254nm) irradiation on egg hatching and adult emergence of the flour beetles, Tribolium castaneum, T. confusum and the almond moth, Cadra cautella. J Insect Sci. 2007;7:1–6. doi: 10.1673/031.007.3601 20233102 PMC2999462

[37] Li M, Yang T, Bui M, Gamez S, Wise T, Kandul NP, et al. Suppressing mosquito populations with precision guided sterile males. Nat Commun. 2021;12(1):5374. doi: 10.1038/s41467-021-25421-w 34508072 PMC8433431

